# Fronto-striatal neurometabolite signatures of impulsivity in early abstinence from methamphetamine

**DOI:** 10.1016/j.nicl.2026.104015

**Published:** 2026-06-01

**Authors:** Alexandru M. Dumitrescu, M. Frances Vest, Jennifer M. Loftis, Kevin S. Murnane

**Affiliations:** a Louisiana Addiction Research Center, Louisiana State University Health Sciences Center at Shreveport, Shreveport, LA, United States; b Department of Pharmacology, Toxicology & Neuroscience, Louisiana State University Health Sciences Center at Shreveport, Shreveport, LA, United States; c Research & Development Service, Veterans Affairs Portland Health Care System, 3710 SW U.S. Veterans Hospital Rd., Portland, OR, United States; d Department of Psychiatry, Center for Mental Health Innovation, Oregon Health & Science University, 3181 SW Sam Jackson Park Rd, Portland, OR, United States; e Department of Behavioral Neuroscience, Oregon Health & Science University, 3181 SW Sam Jackson Park Rd, Portland, OR, United States; f Center for Brain Health, Louisiana State University Health Sciences Center at Shreveport, Shreveport, LA, United States; g Department of Psychiatry and Behavioral Medicine, Louisiana State University Health Sciences Center at Shreveport, Shreveport, LA, United States

**Keywords:** Methamphetamine, Magnetic Resonance Spectroscopy, Neurometabolites, Impulsivity

## Abstract

**Background:**

Methamphetamine use is associated with neurometabolite alterations and increased impulsivity, but relationships between neurometabolites and specific impulsivity dimensions remain unclear, particularly during early abstinence when relapse risk is high and recovery is dynamic.

**Methods:**

We studied 20 recently abstinent individuals and 21 age-matched controls. Single-voxel MRS was acquired in the bilateral dorsolateral prefrontal cortex (DLPFC), medial prefrontal cortex (mPFC), anterior cingulate cortex (ACC), and caudate (CAUD) to quantify N-acetylaspartate (NAA), myo-inositol (mI), choline compounds (GPC + PCh), and glutamate and glutamine (Glu + Gln) concentrations relative to total creatine (Cr + PCr). Group differences in neurometabolite ratios were tested with linear mixed-effects models, and impulsivity was tested across six Barratt Impulsiveness Scale (BIS-11) first-order factors with mixed-design ANCOVA. Regressions assessed whether neurometabolite concentrations predicted impulsivity and whether relationships differed between groups, adjusting for education, depression, anxiety, and stress.

**Results:**

Recently abstinent individuals scored higher on three BIS-11 factors (Motor, Self-Control, Cognitive Complexity). MRS showed elevated mI/Cr + PCr and GPC + PCh/Cr + PCr in the CAUD and increased GPC + PCh/Cr + PCr in the mPFC, while NAA and Glu + Gln ratios did not differ. Motor impulsivity was predicted only by higher stress. Lower self-control was associated with higher CAUD mI/Cr + PCr in age-matched controls but not in recently abstinent individuals. Cognitive Complexity was predicted by group, with no neurometabolite effects.

**Conclusions:**

Fronto-striatal glial dysregulation contributes to impulsivity in methamphetamine use disorder. Preservation of NAA despite glial changes suggests a distinct neurochemical profile of early abstinence. Stress and glial dysfunction, potentially involving neuroinflammatory processes, may jointly drive impulsivity, highlighting intervention targets. Integrating MRS with behavioral measures provides insight into relapse vulnerability.

## Highlights

• Abstinent methamphetamine users showed elevated impulsivity and fronto-striatal glial markers.
• Stress was associated with greater motor impulsivity in the medial prefrontal cortex.
• Caudate myo-inositol predicted poorer self-control in age-matched controls only.

## 1 Introduction

Methamphetamine is a highly addictive psychostimulant known for its long-lasting dysregulating effects on the central nervous system, particularly in brain regions involved in cognition and drug-seeking behavior (London et al., 2015, Nestor et al., 2023). These disruptions are thought to contribute to difficulties with sustained abstinence (Sadek et al., 2007). In the United States, methamphetamine use has increased significantly, becoming a major contributor to drug-related mortality (Hedegaard et al., 2019) with over 2.4 million individuals reporting past-year use by 2024 (SAMHSA, 2025).

At the molecular level, methamphetamine disrupts dopamine, serotonin, and norepinephrine regulation (Carvalho et al., 2012, McKetin et al., 2016; see also Panenka et al., 2013 for review). Chronic exposure results in neuronal loss (Ernst et al., 2000), mitochondrial dysfunction, oxidative stress, and inflammation (Keshavarzi et al., 2019), all contributing to altered brain structure, function, and behavior.

Neuroimaging studies have revealed hippocampal atrophy (Du et al., 2015), gray matter reductions (Nakama et al., 2011), corpus callosum thinning (Du et al., 2015), increased white matter volume (Thompson et al., 2004), and both altered cortical thickness and disrupted network attributes (Luo et al., 2025), as well as functional abnormalities, including reduced D2 receptor availability (Kohno et al., 2018, Volkow et al., 2001a, Volkow et al., 2001b, Volkow et al., 2001c, Volkow et al., 2015). Many of these abnormalities involve fronto-striatal circuits that support executive control and behavioral regulation, systems critically implicated in impulsivity and addiction-related behaviors. Understanding the neurochemical alterations within these circuits may therefore help clarify the biological mechanisms underlying behavioral dysregulation in methamphetamine use disorder.

Magnetic resonance spectroscopy (MRS) provides a non-invasive method to quantify neurochemical changes in vivo. Most MRS studies in individuals with methamphetamine use disorder (abstinent for weeks to months) have focused on N-acetylaspartate (NAA), myo-inositol (mI), choline-containing compounds (GPC + PCh), and the combined signal of glutamate and glutamine (Glu + Gln or Glx), which reflect neuronal viability, neuroinflammation, membrane turnover, and excitatory neurotransmission, respectively (Bakhshinezhad et al., 2022, Howells et al., 2014, Thomson et al., 2024). Far fewer studies have examined methamphetamine-related changes during early abstinence (i.e., the first few weeks).

NAA, a marker of neuronal integrity and viability (Moffett et al., 2007), has consistently shown reductions across multiple brain regions, including the anterior cingulate cortex (ACC), dorsolateral prefrontal cortex (DLPFC), medial prefrontal cortex (mPFC), basal ganglia (BG), and white matter (Chang et al., 2007, Ernst et al., 2000, Howells et al., 2014, Koohsar et al., 2022, Wu et al., 2018), in individuals with methamphetamine use disorder. mI, a glial marker implicated in neuroinflammatory processes and gliosis, is often elevated in stimulant-related neurotoxicity and has been reported in regions like the BG and frontal cortical regions, including the DLPFC, mPFC, and ACC (Ernst and Chang, 2008, Ernst et al., 2000, Nordahl et al., 2005, Nordahl et al., 2002, Salo et al., 2007, Sekine et al., 2002, Sung et al., 2007, Wu et al., 2018). Elevated GPC + PCh reflects cell membrane degradation and glial proliferation, both markers of neural damage (Nordahl et al., 2002, Salo et al., 2011, Salo et al., 2007). Glu + Gln, reflecting excitatory neurotransmission, has shown varied results, with some studies reporting increases (Nordahl et al., 2002, Salo et al., 2011, Salo et al., 2007, Sekine et al., 2002, White et al., 2018, Yang et al., 2018), particularly in the mPFC (Wu et al., 2018), and others noting reductions in dopamine-rich circuits (Koohsar et al., 2022).

In addition to neurochemical alterations, methamphetamine use is associated with cognitive impairments, notably in episodic memory, executive function, processing speed, motor coordination, and visuoconstruction (Grachev et al., 2001, Nordahl et al., 2002, Salo et al., 2011, Scott et al., 2007, Sekine et al., 2002, Vest et al., 2025). These deficits are commonly linked to fronto-striatal neurotoxicity (Wu et al., 2018, Yang et al., 2018) and supported by neuroimaging findings in frontal, striatal, and limbic regions (Jan et al., 2012).

Among affected cognitive domains, impulsivity stands out as a particularly salient and clinically relevant deficit. Impulsivity, characterized by rapid and unplanned reactions without regard to consequences, is a hallmark of methamphetamine use disorder (see also Kozak et al., 2019 for review; Moeller et al., 2001, Yin et al., 2024). It has been consistently linked to poorer treatment outcomes (Lavalley et al., 2025, Moallem et al., 2018) and functional impairment (see also Nordahl et al., 2003, Potvin et al., 2018 for reviews; Wang et al., 2020). Importantly, impulsivity may be particularly pronounced during early abstinence, when withdrawal effects interact with fronto-striatal dysfunction, increasing relapse vulnerability (Jones et al., 2016); yet this stage remains undercharacterized. Impulsivity is often assessed with the Barratt Impulsiveness Scale (BIS-11), a widely used self-report instrument that provides a total score, three second-order factors (Attention, Motor, and Non-Planning), and six first-order factors (i.e., Attention, Cognitive Instability, Motor, Perseverance, Self-Control, and Cognitive Complexity). Most studies have reported elevated total and second-order scores in individuals with a history of methamphetamine use (Cservenka and Ray, 2017, Jones et al., 2016, Yin et al., 2024), especially in Motor and Non-Planning impulsivity (Yin et al., 2024). While the six first-order subscales offer more granular insight, they remain underexplored in stimulant use (Stanford et al., 2009).

Although prior studies have explored neurochemical alterations and neurocognitive deficits, few have examined how these changes relate to impulsivity, especially at the subscale level. This gap limits the understanding of how neurometabolite alterations correspond to specific impulsivity traits (Huang et al., 2020, Jones et al., 2016, Luo et al., 2024, Moallem et al., 2018, Yamamuro et al., 2016). Moreover, little is known about how brain-behavior relationships manifest during early abstinence, a dynamic period when withdrawal-related instability and impulsivity may interact. Investigating these relationships could clarify methamphetamine-related brain dysfunction and guide the development of targeted interventions, particularly given the absence of Food and Drug Administration approved pharmacotherapies for this disorder. To our knowledge, no prior study has simultaneously measured fronto-striatal neurometabolites and BIS-11 subscales in individuals within the first month of methamphetamine abstinence, nor directly tested brain–behavior associations at this granular level of impulsivity traits. By focusing on recently abstinent patients and first-order BIS-11 dimensions, the present study aims to characterize how specific neurometabolic alterations map onto distinct impulsivity components during a period of heightened relapse risk.

We hypothesized that recently abstinent individuals would exhibit reductions of NAA in right and left DLPFC, mPFC, ACC, and CAUD (Chang et al., 2007, Ernst et al., 2000, Howells et al., 2014, Koohsar et al., 2022, Wu et al., 2018), elevated mI (Ernst and Chang, 2008, Ernst et al., 2000, Nordahl et al., 2005, Nordahl et al., 2002, Salo et al., 2007, Sekine et al., 2002, Sung et al., 2007, Wu et al., 2018) and choline (Nordahl et al., 2002, Salo et al., 2011, Salo et al., 2007) in these regions, and altered Glu + Gln levels reflecting disrupted neurotransmission (Nordahl et al., 2002, Salo et al., 2011, White et al., 2018). We also expected that individuals within the first month of abstinence would demonstrate higher impulsivity scores, particularly in Motor and Non-Planning subscales of the BIS-11 (Yin et al., 2024), and that neurometabolite concentrations in fronto-striatal regions would correlate with specific impulsivity traits compared to age-matched controls. Accordingly, our primary objective was not to re-establish these group differences per se, but to determine whether distinct neurometabolic signatures in fronto-striatal circuitry are differentially associated with first-order BIS-11 subscales (e.g., Motor, Perseverance, Self-Control) during early abstinence. Demonstrating such neurometabolite–subscale specificity would provide novel evidence for mechanistic links between regional neurochemical disruption and particular facets of impulsivity that are most relevant to relapse vulnerability in methamphetamine use disorder.

## 2 Materials and methods

### 2.1 Participants

Recruitment was conducted in collaboration with the Council on Alcoholism and Drug Abuse (CADA) of Northwest Louisiana and the UpRising Addiction Center, two residential treatment facilities in the Shreveport-Bossier City area of Louisiana. Twenty individuals (13 men, 7 women; age range: 25–50 years, M = 36 ± 7) were enrolled based on the following criteria: 1) age 25–55; 2) history of methamphetamine use that meets the Diagnostic and Statistical Manual of Mental Disorders 5th edition (DSM-5) criteria for a stimulant use disorder – methamphetamine subtype; 3) minimum 7-day residency at the treatment center; and 4) English fluency. Exclusion criteria included: 1) other substance use disorders (except nicotine or cannabis); 2) inability to understand study materials; and 3) unstable medical/psychiatric conditions, including schizophrenia, Bipolar I disorder (excluding drug-induced states), or history of significant brain injury, stroke, or seizures. To reduce the likelihood of undisclosed recent methamphetamine use, all participants in the methamphetamine group were recruited from abstinence‑oriented residential treatment programs, where they had resided for at least 7 days prior to study enrollment. In both collaborating programs, patients are under close clinical monitoring and urine drug testing is performed when recent substance use is suspected; study visits were coordinated with treatment staff, and individuals with suspected ongoing use were not enrolled. Participants with methamphetamine use disorder were therefore in early abstinence, as determined by residential program enrollment and self‑reported duration of abstinence from methamphetamine. Duration of abstinence was obtained during the in-person screening process, is summarized in Table 1, and averaged 19 ± 4 days at the time of testing. To ensure strict adherence to the inclusion and exclusion criteria, individuals with methamphetamine use disorder underwent evaluation using the Quick Structured Clinical Interview for DSM‑5 Disorders (QuickSCID‑5) (First and Williams, 2022), administered by trained psychiatry residents.

**Table 1:** Demographic and clinical characteristics of age-matched control and methamphetamine groups. Values are presented as mean ± standard deviation (SD) or as number and percentage. Statistically significant between-group differences are indicated as follows: *p* < 0.05 (*), *p* < 0.001 (***). For categorical variables, chi-square or Fisher’s exact tests were used as appropriate, and Cramér’s V was reported as a measure of effect size. *P*-values for depression, anxiety, and stress subscales were adjusted using the Holm–Bonferroni procedure. *N, number of participants.*

	Individuals recently abstinent from methamphetamine (*N* = 20)	Age-matched controls (*N* = 21)	*p*-value	*Cramér’s V* *value*
Demographics				
*Age (years), mean (SD)*	36 ± 7	35 ± 8	0.396	
Sex, N (%)				
*Men*	13 (65%)	12 (57.14%)	0.610	
*Women*	7 (35%)	9 (42.86%)		
Educational level, N (%)			< 0.001^***^	0.79
*Below 8 years*	3 (15%)	−		
*9 – 11 years*	7 (35%)	−		
*High school graduate or GED*	7 (35%)	3 (14.3%)		
*Some college*	1 (5%)	4 (19%)		
*Associate’s degree*	2 (10%)	2 (9.5%)		
*Bachelor’s degree*	−	11 (52.4%)		
*PhD*	−	1 (4.8%)		
Race, N (%)			0.99	0.17
*European American*	17 (85%)	18 (85.7%)		
*African American*	3 (15%)	2 (9.5%)		
*Asian American*	−	1 (4.8%)		
Negative emotional states, mean (SD)				
*Depression*	10.40 ± 9.8	4.5 ± 6.8	0.029*	
*Anxiety*	9.25 ± 7.38	4.65 ± 5.48	0.018*	
*Stress*	14.05 ± 10.50	8.50 ± 5.30	0.044*	
Route of administration, N (%)				
*Inhalation*	11 (55%)	−	−	
*Intravenous*	7 (35%)	−	−	
*Intranasal*	2 (10%)	−	−	
Age of first use				
*Years, mean (SD)*	20 ± 8	−	−	
Duration of use				
*Years, mean (SD)*	15 ± 8	−	−	
Amount used				
*Grams/day, mean (SD)*	2.16 ± 2.98	−	−	
Duration of abstinence				
*Days, mean (SD)*	19 ± 4	−	−	
Co-occurring cannabis use disorder, N (%)				
*Yes*	7 (35%)	−	−	
*No*	13 (65%)	−	−	
Tobacco use, N (%)				
*Yes* *No*	17 (85%) 3 (15%)	14 (66.7%) 7 (33.3%)	0.178	

Twenty-one age-matched controls (12 men, 9 women; age range: 25–47 years, M = 35 ± 8) were recruited from the local community between June 2021 and June 2025. Inclusion criteria were: 1) age 25–55, and 2) English fluency. Exclusion criteria included: 1) meeting DSM-5 criteria for any substance use disorder (except nicotine and cannabis); 2) unstable medical or psychiatric conditions or disorders, including schizophrenia or Bipolar I disorder; 3) history of significant brain injury, stroke, or seizure disorder; and 4) inability to understand the informed consent, study purpose, or procedures.

Psychiatric conditions were defined as prior clinician-diagnosed DSM-5 psychiatric disorders reported by participants. All participants were asked whether they had previously received a formal psychiatric diagnosis. In the methamphetamine group, diagnoses were verified using the QuickSCID-5 to distinguish primary psychiatric disorders from substance-induced acute states. Demographic and clinical data are presented in Table 1.

This study was approved by the Louisiana State University Health Science Center − Shreveport Institutional Review Board. All participants provided written, informed consent in accordance with the Declaration of Helsinki.

An a priori power analysis (G*Power 3.1.9.7) (Faul et al., 2007) indicated that approximately twenty-two participants per group would be required to detect a large effect size (f = 0.40) with 80% power at α = 0.05. The final sample included twenty individuals recently abstinent from methamphetamine and twenty-one age-matched controls, so the methamphetamine group fell slightly below this target.

### 2.2 Clinical and behavioral assessments

#### 2.2.1 Drug use history

To capture the nature of participants' methamphetamine use, they were asked a series of questions regarding their drug use history. These questions included primary route of administration, age of first use, duration of use, amount used per day, and duration of abstinence.

#### 2.2.2 Depression anxiety stress scales – Long Form (DASS-42)

Self-report questionnaires (DASS-42 and BIS-11) were administered individually on a laptop in a quiet, distraction-free room. Details of each instrument are provided below.

The DASS-42 assesses negative emotional states across three domains: depression, anxiety, and stress, with 14 items per subscale (Crawford and Henry, 2003, Lovibond, 1995). Each of the 42 questions is scored on a 4-point Likert scale (0 = Did not apply to me at all to 3 = Applied to me very much, or most of the time). Scores for depression, anxiety and stress are calculated by summing the scores for the relevant items.

#### 2.2.3 Barratt Impulsiveness Scale (BIS-11)

Impulsivity was measured using the 30-item Barratt Impulsiveness Scale (BIS-11) (Patton et al., 1995), administered via Inquisit 5.0 software (De Clercq et al., 2003). Items are rated on a 4-point Likert scale (1 = rarely/never to 4 = almost always/always). While total scores range from 30 to 120, this study focused on six first-order subscales (i.e., Attention, Cognitive Instability, Motor, Perseverance, Self-Control, and Cognitive Complexity) reflecting specific impulsivity traits. Each subscale score is calculated as the sum of its items, yielding ranges of 5–20 for Attention, 2–8 for Cognitive Instability, 7–28 for Motor, 4–16 for Perseverance, 6–24 for Self-Control, and 6–24 for Cognitive Complexity (Patton et al., 1995).

### 2.3 MRS data acquisition and preprocessing

#### 2.3.1 MRS data acquisition

Structural MRI and MRS data were acquired on a Siemens Magnetom Vida 3.0 T scanner (Siemens, Erlangen, Germany) equipped with a 32-channel head coil at Ochsner LSU Health St. Mary’s Medical Center (Shreveport, Louisiana, United States). Three-dimensional T1-weighted images were collected using a magnetization-prepared rapid acquisition gradient echo (MPRAGE) sequence (TR = 2200 ms, TE = 2.4 ms, flip angle = 8°, field of view = 256 × 256 mm^2^, matrix = 256 × 256, 192 slices, slice thickness = 1 mm). MRI acquisition lasted ∼ 5 min.

**Fig. 1:**
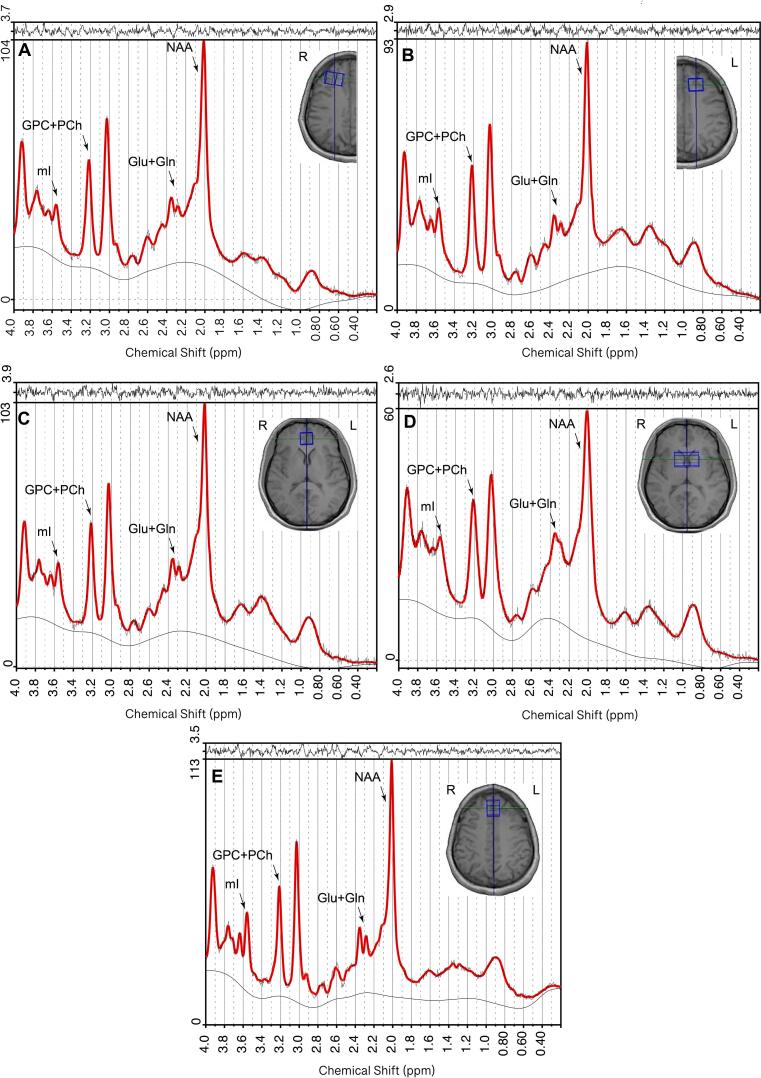
Representative MRS spectra from the five regions of interest (ROIs) in an individual recently abstinent from methamphetamine: A. right dorsolateral prefrontal cortex (DLPFC), B. left DLPFC, C. anterior cingulate cortex (ACC), D. caudate (CAUD), and E. medial prefrontal cortex (mPFC). For each ROI, the acquired spectrum (black line) is overlaid with the LCModel neurometabolite fit (red line). The residual (difference between the measured spectrum and model fit) is shown above each plot. Insets show voxel placement for each ROI. These axial MRI images follow the radiological convention, where the image is horizontally flipped so that the participant’s left hemisphere (L) is displayed on the right side of the image, and the right hemisphere (R) on the left. (For interpretation of the references to colour in this figure legend, the reader is referred to the web version of this article.)

Single-voxel MRS was acquired in five regions of interest (ROIs): left and right DLPFC, ACC, CAUD, and mPFC (Fig. 1), using a short-echo, water-suppressed point-resolved spectroscopy (PRESS) sequence (TR = 2000 ms, TE = 30 ms, 80 averages, spectral bandwidth = 1200 Hz). The voxel size was 20 × 20 × 20 mm^3^ for DLPFC, ACC, and mPFC, and 20 × 40 × 20 mm^3^ for the CAUD. The larger CAUD voxel was selected to increase S/N and ensure adequate coverage of the CAUD while minimizing contamination from surrounding CSF and white matter. Voxel localization followed a standardized protocol using AC–PC–aligned T1-weighted images and anatomical landmarks for each ROI. All voxels were placed by the same trained operator under neuroradiologist supervision, with visual verification in three orthogonal planes to avoid overlap with ventricles, skull, or major vessels. Each MRS voxel acquisition required approximately 3.5 min, totaling 17–18 min for all ROIs. Total scan time (structural MRI + MRS) was ∼ 22–23 min per participant.

#### 2.3.2 Structural MRI processing

T1-weighted MPRAGE images were used to guide MRS voxel placement and to provide anatomical reference for the spectroscopy data. Images were visually inspected for motion and gross artifacts. No automated gray matter (GM), white matter (WM), or cerebrospinal fluid (CSF) tissue segmentation was performed, and no tissue-fraction/partial-volume correction was applied to the MRS neurometabolite estimates. Neurometabolites are reported as ratios to creatine plus phosphocreatine (Cr + PCr) after confirming no group differences in absolute Cr + PCr; this approach reduces inter-individual scaling variability but does not correct for differences in voxel tissue composition.

#### 2.3.3 Preprocessing

MRS spectra were automatically analyzed using the linear combination model implemented in LCModel software (Provencher, 1993) at Louisiana State University Health Sciences Center (Shreveport, United States). Spectral fitting used the simulated 3.0 T PRESS (TE = 30 ms) basis set provided with LCModel and matched to the acquisition sequence. The basis set included the neurometabolites quantified in the present study (NAA, mI, GPC + PCh, Glu + Gln) as well as the standard simulated macromolecule and lipid components supplied with LCModel. Macromolecule and lipid signals were therefore modeled using the default LCModel macromolecule/lipid basis functions rather than being measured experimentally. These components were included only to improve spectral fitting and were not analyzed as outcome variables in the present study.

Spectra were fitted over a 0.2–4.0 ppm range, consistent with recommendations for short-echo PRESS acquisitions. No additional line-broadening or apodization was applied prior to LCModel analysis; linewidth was determined primarily by acquisition quality and subsequently controlled through predefined quality control procedures. Quality control was based on signal-to-noise ratio (S/N) and full width at half maximum (FWHM); only spectra with FWHM ≤ 0.1 ppm and S/N ≥ 20 were retained (Shyu et al., 2021, Wu et al., 2018). Furthermore, only neurometabolite peaks with Cramer-Rao lower bounds (CRLB) < 15% were included in the final analysis (Shyu et al., 2021). These criteria were applied uniformly to NAA, mI, GPC + PCh, and Glu + Gln, and no additional manual outlier removal was performed beyond exclusion of spectra or neurometabolite estimates that failed these prespecified thresholds. Because Glu + Gln arises from J‑coupled resonances with greater spectral overlap than singlet neurometabolites (Merritt et al., 2016), we additionally characterized its CRLB distribution and potential outliers by region and group (Supplementary Tables 3–5; Supplementary Fig. 2).

Neurometabolite concentrations were expressed as ratios to creatine plus phosphocreatine (Cr + PCr), which served as a reference due to its well-established and consistently validated stability across subjects (see Ford and Crewther, 2016 for review). To characterize the behavior of the reference neurometabolites in this sample, we also examined absolute LCModel estimates of Cr + PCr, Cr, PCr, and GPC + PCh across groups and regions (see 2.4.3. MRS group comparisons and Supplementary Tables 1–2) and did not observe any global group differences in these neurometabolites. This normalization minimizes inter-subject variability stemming from technical factors such as coil loading. Reported values include ratios of NAA/Cr + PCr, mI/Cr + PCr, GPC + PCh/Cr + PCr, and Glu + Gln/Cr + PCr.

### 2.4 Statistical analyses

All analyses were conducted using two-tailed tests with α = 0.05. Where applicable*, p-*values for follow-up tests were adjusted for multiple comparisons within each family of tests using Bonferroni or Holm–Bonferroni procedures, as specified in the relevant sections below. All analyses were performed using SPSS version 29.0.2.0 (IBM Corp., Armonk, NY, USA).

#### 2.4.1 Demographic and emotional state comparisons

Age was compared between individuals recently abstinent from methamphetamine (i.e., within the first month of abstinence) and age-matched controls using non-parametric Mann-Whitney U tests. Negative emotional states were assessed using the depression, anxiety, and stress subscales of the DASS-42. Depression and anxiety scores were compared between groups using non-parametric Mann–Whitney U tests, whereas stress scores were analyzed using Welch’s *t*-test due to unequal variances. To control for multiple comparisons across these three related subscales, *p-*values were adjusted using the Holm–Bonferroni method (family-wise α = 0.05).

Sex, tobacco use and co-occurring cannabis use disorder were analyzed using chi-square tests. Educational level and race were analyzed using Fisher’s exact tests due to sparse cell counts and violation of chi-square assumptions (i.e., expected frequencies < 5 in more than 20% of cells or expected counts < 1). Cramér’s V was calculated as a measure of effect size for categorical comparisons. Statistical significance was set at *p* < 0.05.

#### 2.4.2 Impulsivity analysis

A mixed-design ANCOVA was conducted to examine differences in impulsivity across the six first-order subscales of the BIS-11. Population (individuals recently abstinent from methamphetamine vs. age-matched controls) was entered as the between-subjects factor and BIS-11 first-order subscales as the within-subjects factor. Educational level, depression, anxiety, and stress scores were included as covariates to account for their potential influence on impulsivity scores. Pairwise comparisons of estimated marginal means were adjusted using Bonferroni correction. Intercorrelations and collinearity diagnostics for these covariates are reported in Supplementary Table 6.

#### 2.4.3 MRS group comparisons

Group differences in neurometabolite ratios were assessed using linear mixed-effects (LME) models to accommodate the repeated-measures structure of the data, whereby each participant provided measurements from five brain ROIs. LME models were chosen for their ability to model both within-subject (across ROIs) and between-subject (group, covariate) variability, as well as to handle missing data without listwise exclusion. Each model included Population (individuals recently abstinent from methamphetamine vs. age-matched controls), ROI (within-subject factor), and their interaction (Population × ROI) as fixed effects. Educational level and DASS‑42 depression, anxiety, and stress scores were included as covariates in these primary models to account for their potential influence on neurometabolite estimates. Pairwise comparisons of estimated marginal means were adjusted using Bonferroni correction. Intercorrelations and collinearity diagnostics for these covariates are reported in Supplementary Table 6.

Analogous LME models were also fitted for absolute Cr + PCr, Cr, PCr, and GPC + PCh using the same fixed‑effect structure and covariates, with a random intercept for Subject, to assess potential group and regional differences in creatine‑ and choline‑containing neurometabolites (Supplementary Tables 1–2). An unstructured covariance matrix was used to flexibly model variance and covariance across ROIs. Models were estimated by restricted maximum likelihood (REML). This approach provides robust and efficient estimation of group and interaction effects while maintaining statistical power (Bernal-Rusiel et al., 2013, Chen et al., 2013).

#### 2.4.4 ROI-based regression models

Multiple linear regression analyses were performed to examine associations between regional neurometabolite concentrations and specific impulsivity dimensions, as measured with the BIS-11 first-order subscales. For each subscale that exhibited significant group-level differences, a separate regression model was constructed with the subscale score as the dependent variable. Independent variables in each regression were limited to neurometabolite ratios that showed significant group differences, restricted to the brain regions (i.e., ROIs) where those differences were observed. Where appropriate, interaction terms between neurometabolite levels and population group (individuals recently abstinent from methamphetamine vs. age-matched controls) were included to assess potential moderation effects. In all models, educational level, depression, anxiety, and stress scores were included as covariates to account for their potential influence on impulsivity scores. Intercorrelations and collinearity diagnostics are for these covariates are provided in Supplementary Table 6. To account for multiple testing across regression models, *p-*values were adjusted using the Holm–Bonferroni method.

In sensitivity analyses addressing potential multicollinearity among affective covariates, we also derived a composite negative affect score. DASS‑42 depression, anxiety, and stress subscale scores were standardized (z‑scores) across all participants and averaged to create a single negative affect composite indexing overall negative emotional symptoms. In additional LME and regression models, educational level and the negative affect composite were entered as covariates in place of the three separate DASS‑42 subscale scores.

Finally, as an additional sensitivity analysis, exploratory Pearson correlation analyses were performed within the methamphetamine group to examine whether abstinence duration was associated with BIS-11 first-order subscales and ROI-specific neurometabolite ratios that showed significant group differences in the primary analyses. Statistical significance was set at *p* < 0.05.

## 3 Results

### 3.1 Demographic, clinical and impulsivity profile

Table 1 summarizes the demographic and clinical characteristics of both groups. No significant differences were found in age (*p* = 0.396), sex (*p* = 0.610), or race (*p* > 0.05). However, individuals recently abstinent from methamphetamine had significantly lower educational levels, with half reporting 11 years or fewer of education. By contrast, age-matched controls were more likely to have college or graduate degrees.

Individuals recently abstinent from methamphetamine reported significantly higher depression, anxiety, and stress levels, as compared to the age-matched control group. Mann-Whitney U tests showed between-group differences in depression (U = 329.50, p = 0.029) and anxiety (U = 323.00, p = 0.018) levels, while Welch’s *t*-test revealed higher stress levels, t(28.1) = 2.11, p = 0.044. As a result, educational level, depression, anxiety, and stress scores were included as covariates in all subsequent analyses. To characterize relationships among the covariates, we computed Pearson correlations separately by group (Supplementary Table 6). Educational level showed only weak associations with depression, anxiety, and stress in both groups (all |r| ≤ 0.25, all *p* ≥ 0.29). By contrast, depression, anxiety, and stress were strongly intercorrelated in both the methamphetamine group (r = 0.77–0.93, all *p* < 0.001) and age-matched controls (r = 0.63–0.71, all *p* ≤ 0.003). Collinearity diagnostics from the LME models indicated low variance inflation factors (VIFs) for educational level (1.39–1.69) but elevated VIFs for depression (9.55–11.55) and stress (10.61–11.87), consistent with substantial multicollinearity among the emotional-state covariates (Supplementary Table 6).

**Fig. 2:**
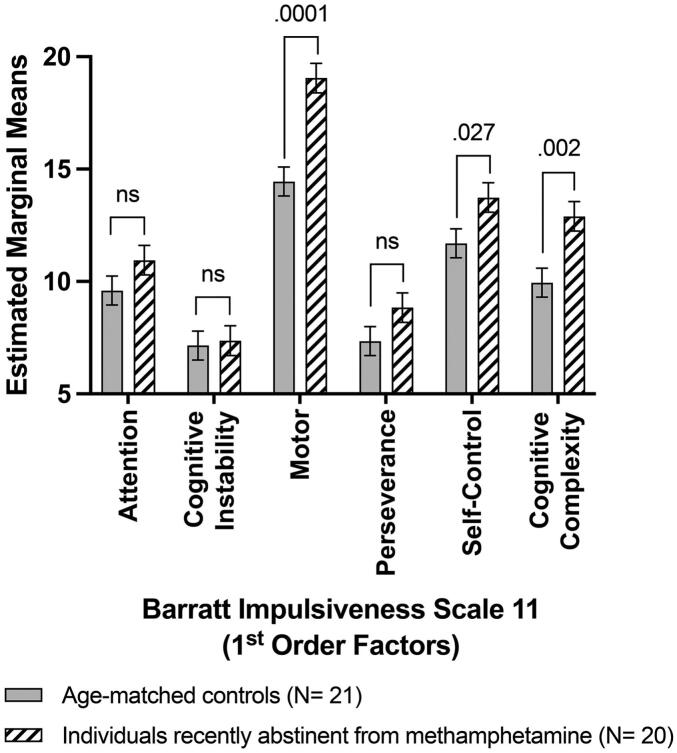
Adjusted marginal means and 95% confidence intervals for BIS-11 first-order subscale scores by group, controlling for educational level, depression, anxiety, and stress scores. Compared to age-matched controls (n = 21), individuals recently abstinent from methamphetamine (n = 20) showed significantly higher scores on the Motor (****p < 0.0001), Self-Control (*p = 0.027), and Cognitive Complexity (**p = 0.002) subscales (*p-*values Bonferroni-corrected for multiple comparisons). No significant group differences were observed for Attention, Cognitive Instability, or Perseverance subscales (ns = not significant, *p* > 0.05).

Using a mixed-design ANCOVA controlling for educational level, depression, anxiety, and stress scores, we found a significant main effect of Population, F(1, 222) = 31.653, p < 0.0001, η^2^ = 0.125, and Subscale, F(5, 222) = 56.200, p < 0.0001, η^2^ = 0.559, along with a significant Population × Subscale interaction, F(5, 222) = 2.723, p = 0.021, η^2^ = 0.058. Follow-up tests indicated higher scores among individuals recently abstinent from methamphetamine on the Motor subscale, F(1, 222) = 25.173, p < 0.0001, η^2^ = 0.102, as well as on Self-Control, F(1, 222) = 4.930, p = 0.027, η^2^ = 0.022, and Cognitive Complexity, F(1, 222) = 10.304, p = 0.002, η^2^ = 0.044. No group differences were found for Attention, Cognitive Instability, or Perseverance (all *p* > 0.10, Fig. 2).

### 3.2 MRS results

LME model analyses revealed neurometabolite- and region-specific group effects after adjusting for educational level, depression, anxiety, and stress scores.

To evaluate the stability of the creatine reference, we examined absolute LCModel estimates of Cr + PCr, Cr, PCr, and GPC + PCh using additional mixed‑effects models. Across all four neurometabolites, there was no main effect of Population (all *p* ≥ 0.46), indicating an absence of global group differences in their absolute levels. Strong main effects of Region were present for each neurometabolite. Population × Region interactions were observed for Cr + PCr and Cr, reflecting modest region-specific variability rather than systematic differences between groups, and interactions were not significant for PCr and GPC + PCh. Region‑wise group differences in absolute values were small relative to within‑group variability (Supplementary Tables 1–2), supporting the use of Cr + PCr as a relatively stable internal reference in this cohort.

**Fig. 3:**
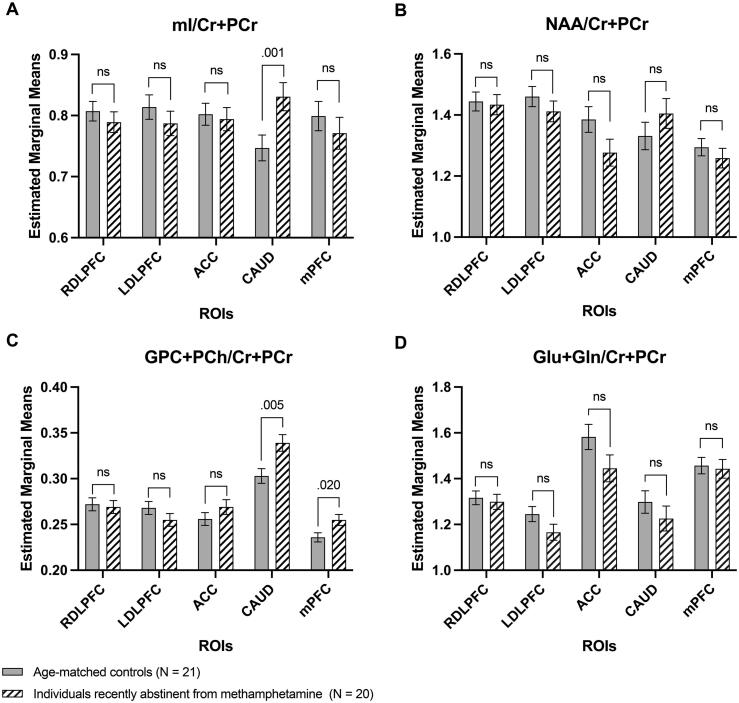
Adjusted marginal means and 95% confidence intervals for neurometabolite concentration ratios by group and region of interest (ROI), controlling for educational level, depression, anxiety, and stress scores. A. mI/Cr + PCr, B. NAA/Cr + PCr, C. GPC + PCh/Cr + PCr, D. Glu + Gln/Cr + PCr. All pairwise group comparisons are Bonferroni-corrected for multiple comparisons. (ns = not significant, *p* > 0.05).

mI/Cr + PCr ratio. No main effect of Population was found, *F*(1, 34.56) = 0.001, *p* = 0.977, but the Population × ROI interaction was significant, *F*(4, 35.05) = 4.090, *p* = 0.008, indicating region-specific group differences. Post hoc tests showed significantly higher mI/Cr + PCr levels in the CAUD of individuals recently abstinent from methamphetamine (*p* = 0.001), with no group differences in the ACC (*p* = 0.654), left DLPFC (*p* = 0.623), mPFC (*p* = 0.726), or right DLPFC (*p* = 0.726; Fig. 3A). Anxiety was positively associated with mI/Cr + PCr levels across participants (*p* = 0.003), consistent with a possible relationship involving glial metabolism.

NAA/Cr + PCr ratio. No significant main effect of Population, *F*(1, 34.38) = 0.532, *p* = 0.471, or Population × ROI interaction, *F*(4, 36.11) = 1.332, *p* = 0.277, was observed. However, a strong main effect of ROI emerged, *F*(4, 36.12) = 13.291, *p* < 0.0001, indicating regional differences in NAA (Fig. 3B). Anxiety was positively associated with NAA/Cr + PCr levels (p = 0.005), consistent with a potential relationship involving neuronal metabolism.

GPC + PCh/Cr + PCr ratio. There was no significant main effect of Population, F(1, 34.90) = 2.271, p = 0.141, but a significant Population × ROI interaction was observed, F(4, 33.74) = 3.047, p = 0.030. Post hoc tests showed higher GPC + PCh/Cr + PCr levels in the CAUD (*p* = 0.005) and mPFC (*p* = 0.020) of the individuals recently abstinent from methamphetamine relative to age-matched controls, with no differences in the ACC (*p* = 0.895), left DLPFC (*p* = 0.281), or right DLPFC (*p* = 0.193; Fig. 3C).

Glu + Gln/Cr + PCr ratio. No significant main effect was found for Population, F(1, 31.20) = 2.353, p = 0.135, or Population × ROI interaction, F(4, 35.61) = 0.792, p = 0.538. A robust main effect of ROI was observed, F(4, 35.61) = 27.838, p < 0.0001, indicating regional Glu + Gln/Cr + PCr variation without group differences (Fig. 3D). For Glu + Gln/Cr + PCr, the subset of estimates that passed QC exhibited mean CRLB values of approximately 6–7% across ROIs and groups, with medians of 6–7% and ranges largely between 4% and 11% (up to 14% in a few ACC and mPFC cases; Supplementary Table 3). Formal comparisons of Glu + Gln CRLB between groups revealed no significant differences in any ROI (all *p* ≥ 0.14; Supplementary Table 4), and only a small number of mild high‑CRLB outliers were detected per ROI and group, with no estimates exceeding 15% CRLB (Supplementary Table 5; Supplementary Fig. 2).

### 3.3 BIS-11 impulsivity subscales and neurometabolite ratio correlations

Analyses focused on BIS-11 subscales showing group differences (i.e., Motor, Self-Control, Cognitive Complexity) and on brain regions with significant neurometabolite differences (i.e., CAUD, mPFC). All regression models included population group, educational level, depression, anxiety, and stress scores as covariates. Neurometabolite × group interaction terms were added where applicable to test for moderation effects.

#### 3.3.1 Motor impulsivity

In the CAUD, a multiple linear regression with Motor impulsivity as the outcome and mI/Cr + PCr, GPC + PCh/Cr + PCr, group, and interaction terms as predictors yielded a significant overall model, *F*(9, 27) = 3.75, *p* = 0.004, explaining 55.5% of the variance (adjusted *R^2^* = 0.407), though no individual predictors reached significance (all *ps* > 0.13).

In the mPFC, the model including GPC + PCh/Cr + PCr, group, and covariates was also significant, *F*(7, 28) = 6.43, *p* < 0.001, explaining 61.6% of the variance (adjusted *R^2^* = 0.520). Only stress significantly predicted Motor impulsivity (β = 0.630, *p* = 0.028); no neurometabolite levels, group, or interaction effects were significant.

#### 3.3.2 Self-Control

**Fig. 4:**
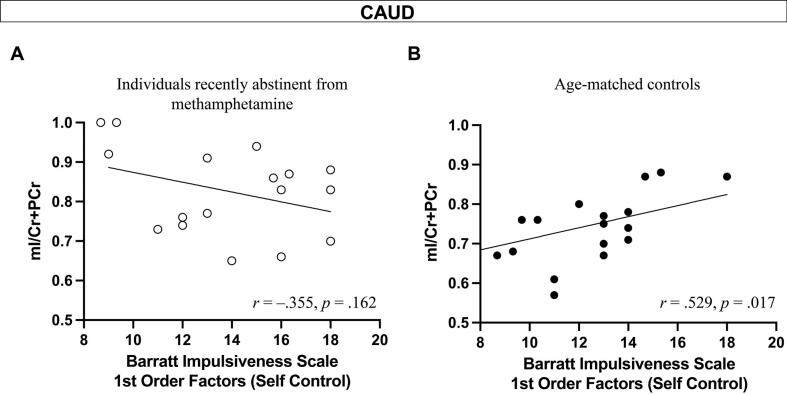
Relationship between ml/Cr + PCr ratio in the caudate and BIS-11 Self-Control subscale scores by group. A. In individuals recently abstinent from methamphetamine, the relationship was nonsignificant and negative, whereas B. in age-matched controls, higher mI/Cr + PCr ratio was associated with higher Self-Control scores (i.e., higher impulsivity). *P-*values shown are Holm–Bonferroni-corrected within the family of neurometabolite–impulsivity tests.

In the CAUD, the regression model including mI/Cr + PCr, GPC + PCh/Cr + PCr, group, and interaction terms was significant, *F*(9, 27) = 2.31, *p* = 0.045, explaining 43.5% of the variance in Self-Control scores (adjusted *R^2^* = 0.246). Significant predictors included group (β = –4.76, *p* = 0.026) and the mI/Cr + PCr × group interaction (β = 2.62, *p* = 0.036). Follow-up analyses showed that in age-matched controls, higher CAUD mI/Cr + PCr was associated with higher self-control scores (β = 0.529, *p* = 0.017; Fig. 4A), which on the BIS-11 indicates poorer self-control (i.e., greater impulsivity). In contrast, this association was nonsignificant and in the opposite direction among individuals recently abstinent from methamphetamine (β = –.355, *p* = 0.162; Fig. 4B).

In the mPFC, the model with GPC + PCh/Cr + PCr, group, and covariates was also significant, *F*(7, 28) = 3.26, *p* = 0.012, accounting for 44.9% of the variance (adjusted *R^2^* = 0.311). No predictors reached significance (all *ps* > 0.08), though trends were noted, and no covariates significantly predicted Self-Control.

#### 3.3.3 Cognitive Complexity

In the CAUD model, the regression was significant, *F*(9, 27) = 2.53, *p* = 0.030, explaining 45.8% of the variance in Cognitive Complexity scores (adjusted *R^2^* = 0.277). A trend toward a group effect was observed (β = –3.90, *p* = 0.059), but no significant effects emerged for neurometabolite predictors or interactions.

In the mPFC, the model was also significant, *F*(7, 28) = 4.16, *p* = 0.003, accounting for 51.0% of the variance (adjusted *R^2^* = 0.387). A significant group effect was found (β = –3.59, *p* = 0.038), with a trend toward a neurometabolite × group interaction (β = 3.02, *p* = 0.066). Follow-up analyses showed no significant association between GPC + PCh/Cr + PCr and Cognitive Complexity in individuals recently abstinent from methamphetamine (β = –.154, p = 0.569; Supplementary Fig. 1A), as well as a non-significant positive trend in age-matched controls (β = 0.424, p = 0.063; Supplementary Fig. 1B).

In sensitivity analyses, we re-estimated the mixed-design ANCOVA and regression models using education and the negative affect composite as covariates instead of the separate DASS-42 depression, anxiety, and stress scores. Motor impulsivity and Cognitive Complexity group differences, as well as the overall pattern of MRS group effects, were replicated, whereas the group difference in Self-Control and the CAUD mI/Cr + PCr–Self-Control association in age-matched controls were attenuated and no longer statistically significant; similarly, the trend-level association between Cognitive Complexity and mPFC GPC + PCh/Cr + PCr in controls was no longer evident (Supplementary Table 7).

Finally, exploratory sensitivity analyses within the methamphetamine group revealed no significant associations between abstinence duration and Motor impulsivity, Self-Control, or Cognitive Complexity scores (all *p* > 0.05). Similarly, abstinence duration was not significantly associated with CAUD mI/Cr + PCr, CAUD GPC + PCh/Cr + PCr, or mPFC GPC + PCh/Cr + PCr ratios (all *p* > 0.05; Supplementary Table 8).

## 4 Discussion

This study investigated neurometabolite changes in fronto-striatal regions and their relationships with impulsivity dimensions in individuals recently abstinent from methamphetamine, focusing on the earliest weeks of abstinence seldom examined in prior MRS studies. Key findings include: (1) elevated myo-inositol and choline in the CAUD and mPFC in the methamphetamine group, as compared to the age-matched control group; (2) higher impulsivity scores in the domains of Motor, Self-Control, and Cognitive Complexity in the methamphetamine group, as compared to the age-matched control group; and (3) neurometabolite-impulsivity associations were influenced by stress and anxiety across both groups. In sensitivity analyses using a composite negative affect covariate, Motor and Cognitive Complexity differences were replicated, whereas the Self-Control difference was attenuated.

### 4.1 Elevated and selective impulsivity dimensions after methamphetamine abstinence

Consistent with prior research (Cservenka and Ray, 2017, Jones et al., 2016, Yin et al., 2024), individuals recently abstinent from methamphetamine showed elevated scores on three BIS-11 subscales: Motor impulsivity, Self-Control, and Cognitive Complexity. Notably, Motor and Cognitive Complexity group differences remained significant when we used a composite negative affect covariate, whereas the Self-Control difference was reduced to a non‑significant trend, suggesting that the latter effect is more sensitive to how affective symptoms are modelled. Comparative studies demonstrate that impulsivity is a pronounced feature both during active methamphetamine use and in the earliest stages of abstinence, with self-reported impulsivity increasing within the first week after cessation compared to active use (Jones et al., 2016). This suggests impulsivity impairments emerge rapidly and may contribute to relapse during recovery (Jones et al., 2016). However, current longitudinal data do not clarify whether these elevated levels persist for several months or how rapidly partial recovery occurs with long-term abstinence (Jones et al., 2016). Evidence from impulse inhibition tasks suggests that significant deficits can last up to 10 months following cessation, with substantial improvement seen after extended abstinence (Liu et al., 2021). Further studies are needed to delineate the trajectory of these impulsivity changes over the full abstinence timeline. These findings reflect the multidimensional nature of impulsivity and its disruption in stimulant use disorders, particularly in domains related to behavioral regulation and executive functioning (see also Kozak et al., 2019 for review; Moeller et al., 2001). Importantly, additional factors such as sleep disturbances (Wang et al., 2024), level of drug craving (Yang et al., 2025), and cortical morphology and network alterations (Luo et al., 2025) may further interact with impulsivity and exacerbate vulnerability during early abstinence.

Elevated Motor impulsivity, defined as acting quickly or without deliberation, is strongly tied to poor inhibition and increased risk-taking (Bari and Robbins, 2013; see also Jupp and Dalley, 2014 for review; Moeller et al., 2001, Monterosso et al., 2005). Higher Self-Control scores defined as reduced capacity for planning and restraint, reflect greater impulsivity and difficulties with goal-directed behavior (London et al., 2004, Nestor et al., 2011). Cognitive Complexity reflects a preference for and ability to engage in complex problem-solving and flexible thinking; however, higher Cognitive Complexity scores in this context may instead indicate difficulties with cognitive flexibility and abstract reasoning (see Dean et al., 2013, Scott et al., 2007 for reviews).

Notably, Perseverance, defined as the capacity to persist with tasks or routines over time, did not differ between groups. This was consistent with mixed findings from the Wisconsin Card Sorting Test (WCST), where some studies report increased perseverative errors (Cox et al., 2016, Farhadian et al., 2017, Han et al., 2008, Hosak et al., 2012) and others do not (see also Hart et al., 2012 for review; Hosak et al., 2012, Karabulut, 2023). This suggests self-report and behavioral markers capture distinct mechanisms (see also Hart et al., 2012 for review; Hosak et al., 2012, Karabulut, 2023), warranting integration with reversal learning or set-shifting tasks.

Similarly, no group differences were found in Attention, defined as the ability to maintain focus on tasks and resist distraction, or Cognitive Instability, defined as a tendency toward racing thoughts and shifting focus. This supports the view that methamphetamine-related impairments are more pronounced in action regulation and planning than attention. These findings align with prior reports that Motor and Non-Planning impulsivity are typically elevated across substance use disorders, whereas attentional impulsivity is not (Moeller et al., 2001). Such results highlight the clinical utility of examining BIS-11 subscales independently, rather than relying on total scores (Stanford et al., 2009).

Clinically, increased Motor and Non-Planning impulsivity have been linked to treatment dropout, relapse risk, and poor adherence (del Palacio-Gonzalez et al., 2024, Pepe et al., 2023, Winhusen et al., 2013). Therefore, interventions targeting these impulsivity dimensions may be critical to improving outcomes. Findings from (Vest et al., 2025) showed that individuals recently abstinent from methamphetamine demonstrate selective deficits in response inhibition, evidenced by longer stop signal reaction times in the Stop Signal Task (SST) and lower Stroop accuracy. In contrast, risk taking and delay sensitivity, as measured by the Balloon Analogue Risk Task (BART) and Iowa Gambling Task (IGT), respectively, were not significantly altered compared to age-matched controls. This identifies response inhibition as a central therapeutic target and emphasizes the value of interventions focused on enhancing executive control and self-regulation to reduce impulsivity-driven dropout and relapse in methamphetamine use disorder.

### 4.2 Region- and neurometabolite-specific alterations

#### 4.2.1 Region-specific glial alterations following methamphetamine abstinence

As hypothesized, we observed region-specific neurometabolite alterations in individuals recently abstinent from methamphetamine, consistent with glial metabolic alterations and processes that may involve neuroinflammation. Gliotic signatures appeared within the first weeks, suggesting early glial dysregulation that may reflect, or contribute to, neuroinflammatory processes and hinder recovery.

In the CAUD, a dopamine-rich region for reward and habit formation (Chang et al., 2005b, McConnell et al., 2015), both mI/Cr + PCr and GPC + PCh/Cr + PCr ratios were significantly elevated. In the mPFC, only GPC + PCh/Cr + PCr was elevated, highlighting fronto-striatal vulnerability to methamphetamine-related neurotoxicity (Nordahl et al., 2002, Salo et al., 2011, Wu et al., 2018). These elevations suggest glial proliferation and membrane turnover (Chang et al., 2005a, McConnell et al., 2015, Nordahl et al., 2002, Wu et al., 2018) consistent with prior MRS, Positron Emission Tomography (PET) and postmortem evidence of microglial activation in dopamine-innervated regions (Howells et al., 2014, Sekine et al., 2002). This interpretation is supported by PET evidence of markedly elevated activated-microglia signal in abstinent methamphetamine users that scales inversely with abstinence duration, consistent with a prominent early-abstinence neuroimmune component (Sekine et al., 2008).

Mechanistically, methamphetamine-induced molecular cascades involving TNF-α signaling, NF-κB activation, and upregulation of matrix metalloproteinase-9 (MMP-9) may compromise blood–brain barrier integrity and increase proinflammatory cytokine release (E Cisneros and Ghorpade, 2012; Jones et al., 2021; see also Loftis and Janowsky, 2014 for review), mechanisms that may underlie the neurometabolite alterations observed in this study. Preclinical work also supports roles for astrocytic hypertrophy and glial fibrillary acidic protein (GFAP) upregulation in methamphetamine-related neuroinflammatory responses (Tehrani et al., 2019). However, because we did not measure peripheral or molecular inflammatory markers, these interpretations should be viewed as indirect and hypothesis‑generating rather than definitive evidence of neuroinflammation.

Higher anxiety levels were also associated with elevated mI/Cr + PCr, aligning with prior links between mood disturbances and glial reactivity (Brunst et al., 2019, Haroon et al., 2017, Sanacora and Banasr, 2013). This supports an integrative model where affective and neurochemical dysregulation co-occur in stimulant use disorders (Hsieh et al., 2014). Notably, depression, anxiety, and stress scores were strongly intercorrelated, and VIFs for depression and stress were high, indicating multicollinearity among these measures. Thus, while including them as covariates helps account for affective differences between groups, it likely inflates standard errors and reduces power to detect small neurometabolite and brain–behavior effects. In addition, alcohol use was not systematically assessed in this sample, so unmeasured differences in alcohol consumption could have influenced neurometabolite levels and impulsivity measures and cannot be ruled out as a potential confound (Gomez et al., 2012, Jiang et al., 2013, Perica et al., 2026, Porter et al., 2025, Schweinsburg et al., 2003).

Notably, glial alterations were regionally specific: both mI/Cr + PCr and GPC + PCh/Cr + PCr were elevated in the CAUD, while only GPC + PCh/Cr + PCr was increased in the mPFC. No changes were observed in the ACC or DLPFC, suggesting regional variability in glial responsiveness or recovery trajectories, potentially influenced by neurotransmitter innervation patterns or stress sensitivity. Similar regional susceptibilities have been observed in HIV + populations, highlighting the translational relevance of these findings (Chang et al., 2005b).

Overall, these results indicate persistent gliotic signatures in fronto-striatal circuits critical for motivation, emotion, and executive control, likely contributing to dysfunction during abstinence.

#### 4.2.2 Widespread glutamatergic dysregulation and preserved neuronal integrity during early methamphetamine abstinence

In contrast to the region-specific glial changes, Glu + Gln/Cr + PCr levels exhibited significant regional variation across ROIs but no significant population effect, suggesting that excitatory metabolism may be broadly distributed across regions rather than showing region-specific alterations between groups. This pattern may be consistent with alterations in glutamate-glutamine cycling reported in prior methamphetamine studies (Glasner-Edwards et al., 2010, Su et al., 2017). Although some studies report Glx/Cr + PCr increases (Nordahl et al., 2002, Salo et al., 2011, Salo et al., 2007, Sekine et al., 2002, White et al., 2018, Yang et al., 2018), our findings align with early reductions that may normalize over time (see Hellem et al., 2015 for review). At the same time, short‑echo Glu + Gln quantification at 3.0 T is inherently challenging because of J‑coupling and overlap with neighboring resonances, and Glu + Gln estimates tend to have higher CRLB and lower precision than NAA or choline compounds (Merritt et al., 2016). Although our supplementary QC analyses indicate acceptable Glu + Gln CRLB values and comparable fit quality between groups, it remains possible that very subtle Glu + Gln differences were not detected due to these sensitivity constraints.

NAA/Cr + PCr levels did not differ between groups, contrasting with studies reporting NAA decreases after longer abstinence (Chang et al., 2007, Ernst et al., 2000, Howells et al., 2014, Koohsar et al., 2022, Wu et al., 2018). One explanation is abstinence stage: NAA reductions may emerge later, reflecting gradual neuronal compromise, while glial markers arise earlier. Thus, glial activation manifests rapidly, whereas neuronal integrity markers initially remain stable initially (Re et al., 2022, Yang et al., 2018, Yoon et al., 2010).

Even after accounting for covariates, Glx/Cr + PCr reductions persisted. Anxiety was positively associated with both NAA/Cr + PCr and mI/Cr + PCr, suggesting emotional distress may modulate neurometabolic markers independent of drug exposure (Abdallah et al., 2013, Dehdar and Raoufy, 2023, Maron and Nutt, 2017). This supports links between anxiety, microglial activation, and glutamatergic imbalance (Beitchman et al., 2020, Dehdar and Raoufy, 2023, Peng et al., 2024, Sah and Singewald, 2025).

Taken together, the combination of elevated glial‑related markers (mI and GPC + PCh) in the CAUD and mPFC, reduced Glu + Gln across regions, and preserved NAA suggests a staged pattern of methamphetamine‑related neuroadaptation in early abstinence. In dopamine‑rich fronto‑striatal circuits, repeated methamphetamine exposure may trigger microglial and astrocytic activation and membrane turnover (reflected in higher mI and GPC + PCh), while compensatory downregulation of glutamatergic tone or impaired glutamate–glutamine cycling (reflected in lower Glu + Gln) serves to limit further excitotoxic damage. The absence of NAA reductions indicates that, at this early abstinence stage, neuronal integrity is relatively preserved, and neurometabolic changes are dominated by glial and synaptic processes rather than frank neuronal loss, in line with longitudinal work showing that structural and NAA changes often emerge later in the course of stimulant use.

### 4.3 Impulsivity and neurometabolite associations

Although Motor impulsivity was higher in individuals with a history of methamphetamine use, it was not directly associated with CAUD or mPFC neurometabolite levels. Motor impulsivity, however, was predicted only by higher levels of stress. Neither neurometabolite levels measured in the mPFC nor differences in experimental groups (individuals recently abstinent from metahmphetamine vs. age-match controls) were significant predictors of impulsive actions. In other words, among all the factors assessed, only stress level showed a meaningful link to impulsive action, while other brain chemistry variables and group identity did not influence this behavior. This suggests that stress-related impulsivity may occur independently of substance use (see also Arnsten, 2009 for review; Arnsten, 2015, Shields et al., 2016, Sinha, 2008).

For Self-Control, exploratory analyses in the primary models suggested that higher CAUD mI/Cr + PCr in age-matched controls was associated with poorer self-regulation, possibly reflecting glial roles in executive processes (Rae, 2014). This link was absent in the methamphetamine group, perhaps due to trait-level vulnerability factors (e.g., genetics, early adversity, chronic exposure) obscuring proximal neurochemical–behavioral associations (Chang et al., 2024, Ducci and Goldman, 2012, Elam et al., 2016, Gerring et al., 2024, Ko et al., 2024; see also Kozak et al., 2019 for review). However, in sensitivity analyses using education and the negative affect composite as covariates, both the group difference in Self-Control and the CAUD mI/Cr + PCr–Self-Control association in age-matched controls were attenuated and no longer reached statistical significance, and the trend-level association between Cognitive Complexity and mPFC GPC + PCh/Cr + PCr in age-matched controls also disappeared. Thus, glial–behavior associations detectable in some specifications should be interpreted cautiously and considered hypothesis-generating rather than definitive. These results nevertheless highlight the value of integrating biological markers with psychiatric history and genetic risk when investigating impulsivity in addiction.

Acute withdrawal, stress activation, and neuroinflammation may further obscure such links early in abstinence.

### 4.4 Clinical implications

The present pattern of preserved NAA with altered fronto-striatal glial-related markers and reduced Glu + Gln during early abstinence, together with stress-related but only modest neurometabolite–impulsivity associations, suggests that this period represents a window in which neural integrity is still largely maintained but glial and synaptic signaling are dysregulated. This interpretation is consistent with longitudinal neuroimaging and MRS studies showing that early abstinence is characterized by dynamic neurochemical changes and partial metabolic recovery over the first weeks, including normalization of NAA, glutamate, and choline levels alongside improvements in cognitive function (Bartsch et al., 2007, Mon et al., 2012). Clinically, this supports targeting interventions to the first weeks of abstinence that aim to normalize glutamatergic signaling and related neurobiological processes (e.g., anti-inflammatory or glia-modulating agents and glutamatergic modulators) while simultaneously addressing stress-related impulsivity through cognitive-behavioral strategies focused on response inhibition and emotion regulation. Consistent with this perspective, pharmacological trials have shown that treatment-related changes in glutamatergic and GABAergic metabolites measured with MRS can track abstinence outcomes, supporting glutamatergic modulation as a potential therapeutic mechanism and biomarker target (Prisciandaro et al., 2021).

The dissociation between robust elevations in Motor, Self-Control, and Cognitive Complexity impulsivity and relatively subtle fronto-striatal neurometabolite–impulsivity associations also suggests that stress and other environmental or developmental factors may be proximal drivers of risky decision-making in this stage. Indeed, previous neuroimaging studies have reported that stress-related dysfunction in prefrontal–striatal circuits during early treatment entry predicts subsequent heavy drinking or relapse risk (Blaine et al., 2020), while MRS studies indicate that large behavioral differences in impulsivity can coexist with relatively modest metabolite–impulsivity coupling (Porter et al., 2025). From a treatment perspective, integrating stress-reduction approaches (e.g., mindfulness-based or trauma-informed interventions) with intensive relapse-prevention around early abstinence may therefore be particularly important, as these approaches have been shown to reduce relapse and heavy substance use in clinical trials (Bowen et al., 2014, Rockhill et al., 2025). In this context, MRS-derived neurometabolite measures may serve as candidate biomarkers for monitoring treatment response in future parmacological and behavioral intervention studies (Prisciandaro et al., 2021, Prisciandaro et al., 2025).

### 4.5 Limitations and future research

This study has several limitations. The modest sample size, though powered to detect large effects, limits generalizability and prevented subgroup analyses. Its cross-sectional design precludes causal inference, and the focus on fronto-striatal regions excluded other areas implicated in impulsivity (e.g., orbitofrontal cortex, insula). Methamphetamine use history relied on self-report without systematic biological verification (e.g., urine screening), and although abstinence duration was relatively homogeneous in this cohort (19 ± 4 days), it was not included as a primary covariate in the main statistical models. Nevertheless, exploratory sensitivity analyses within the methamphetamine group did not reveal significant associations between abstinence duration and the primary impulsivity or neurometabolite findings, suggesting that the observed effects were not strongly driven by small differences in abstinence duration within this early abstinence window. Larger longitudinal studies spanning broader abstinence intervals will nevertheless be necessary to characterize temporal recovery trajectories more precisely. In addition, contributors such as early trauma or genetic vulnerability were not assessed. Furthermore, because nicotine and cannabis use were permitted in both groups and alcohol use was not systematically quantified, the potential contribution of other substances to the observed neurometabolite and impulsivity findings cannot be fully excluded. Future studies with larger samples and more comprehensive characterization of polysubstance use will be important to further disentangle methamphetamine-specific effects from the potential influence of other substances. Importantly, all individuals in the methamphetamine group were recruited from abstinence-oriented residential programs with at least seven days of supervised residence before enrollment, where patients are closely monitored and undergo urine drug testing when recent substance use is suspected. Study visits were also coordinated with treatment staff to reduce the likelihood of undisclosed recent methamphetamine use.

The methamphetamine group size was slightly below the a priori target, which may reduce power to detect small effects or higher-order interactions. Nevertheless, the use of mixed-design ANCOVA and linear mixed-effects models with repeated measures, together with the medium-to-large effect sizes observed for key behavioral and neurometabolite outcomes, suggests that the primary findings are robust.

Methodological limitations related to MRS acquisition should also be considered. We did not perform GM/WM/CSF segmentation of the MRS voxels and therefore did not apply tissue-fraction (partial-volume) correction. As a result, inter-individual or group differences in voxel tissue composition and region-specific voxel geometry may contribute to variability in neurometabolite estimates. Although we minimized these effects through standardized voxel placement, consistent voxel size per ROI, and strict spectral quality control, residual tissue-composition effects may remain. Reporting neurometabolites as Cr + PCr ratios reduces scaling variability but is not a substitute for tissue-fraction correction.

In addition, the short-echo PRESS protocol and basis set used in this study did not allow reliable quantification of GABA and NAAG, preventing the calculation of a GABA/NAAG ratio (Bhogal et al., 2017, Zöllner et al., 2026). Future studies using GABA-optimized acquisition protocols will be necessary to investigate inhibitory neurotransmission more directly.

A further limitation relates to the statistical modeling of group differences. Educational level and DASS-42 depression, anxiety, and stress scores differed between groups and were therefore included as covariates in all models. Although education showed only weak correlations with the emotional variables, depression, anxiety, and stress were strongly intercorrelated, and collinearity diagnostics revealed elevated VIFs for depression and stress. This multicollinearity likely reduced the precision of regression coefficients and the sensitivity to detect smaller neurometabolite or impulsivity effects, particularly given the modest sample size. Consistent with this interpretation, some impulsivity findings—most notably the Self-Control group difference, the CAUD mI/Cr + PCr–Self-Control association, and the mPFC GPC + PCh/Cr + PCr–Cognitive Complexity trend in age-matched controls—were attenuated in sensitivity analyses using a composite negative-affect covariate. These results should therefore be interpreted as exploratory and in need of replication. In this context, the significant findings may be considered conservative, whereas null or borderline results for subtle effects should be interpreted cautiously and require confirmation in larger cohorts.

Another limitation is that alcohol consumption was not systematically recorded, preventing us from modeling its potential effects on neurometabolite concentrations and impulsivity. Future studies should incorporate detailed assessment of alcohol use and other commonly co-used substances to better characterize their potential contribution to brain–behavior relationships in methamphetamine use disorder (Gomez et al., 2012, Jiang et al., 2013, Perica et al., 2026, Porter et al., 2025, Schweinsburg et al., 2003).

Future research should employ longitudinal designs, incorporate objective drug-use verification measures, and expand neuropsychological and genetic assessments. Larger multimodal investigations combining MRS with complementary techniques such as PET and diffusion tensor imaging could help clarify how neurometabolic alterations interact with network-level structural and molecular changes. Such approaches may provide a more comprehensive understanding of the mechanisms underlying impulsivity deficits in methamphetamine use disorder and inform targeted interventions, including behavioral therapies, aerobic exercise (Lyu et al., 2025), or neuromodulation strategies.

## 5 Conclusion

The present findings indicate that fronto-striatal glial alterations play a key role in the increase impulsivity observed in methamphetamine use disorder. The preservation of neuronal integrity markers such as NAA, despite glial abnormalities, points to a unique neurochemical signature during early abstinence. Interactions between stress-related mechanisms and glial alterations, potentially involving neuroinflammatory pathways, may underlie these behavioral manifestations, identifying potential targets for therapeutic intervention. Combining MRS-derived neurometabolite indices with behavioral assessments offers a valuable framework for understanding relapse risk and guiding recovery strategies.

## CRediT authorship contribution statement

**Alexandru Mihai Dumitrescu:** Writing – original draft, Visualization, Validation, Software, Methodology, Investigation, Formal analysis, Data curation, Conceptualization. **M. Frances Vest:** Writing – review & editing, Methodology, Investigation, Conceptualization. **Jennifer M. Loftis:** Writing – review & editing. **Kevin S. Murnane:** Writing – review & editing, Supervision, Project administration, Investigation, Funding acquisition, Conceptualization.

## Funding

This study was supported by the National Institute on Drug Abuse of the National Institutes of Health under grants R01DA061433, UG3DA067299, and R41DA059296, as well as by the Louisiana Addiction Research Center Opioid Settlement Program funded by Caddo Parish. Additional support was provided by the Center for Cardiovascular Diseases and Sciences, the Department of Pharmacology, Toxicology & Neuroscience, the Louisiana Addiction Research Center, and LSU Health Sciences Center at Shreveport.

This material is the result of work supported with resources and the use of facilities at the VA Portland 10.13039/100018696Health Care System (VAPORHCS) and 10.13039/100006668Oregon Health and Science University (OHSU), Portland, OR. This work was funded, in part, by grants from the 10.13039/100000002National Institutes of Health (#R41DA059296-01A1 and #R01MH124824) and the 10.13039/100000738United States Department of Veterans Affairs Clinical Sciences 10.13039/100006190Research and Development Merit Review Program (#I01 CX002668).

The work was conducted using facilities at the Veterans Affairs Portland Health Care System and Oregon Health & Science University, Portland, OR, USA. The contents do not represent the views of the U.S. Department of Veterans Affairs or the United States Government.

## Declaration of competing interest

The authors declare that they have no known competing financial interests or personal relationships that could have appeared to influence the work reported in this paper.

## Data Availability

The raw data supporting the conclusions of this article are not publicly available due to ethical and legal restrictions related to participant privacy and institutional policy. De-identified data may be made available to qualified researchers upon reasonable request, contingent on approval from the study’s principal investigator and compliance with LSU Health Shreveport policies. This includes the potential establishment of a Collaborative Research Agreement or Material Transfer Agreement. Requests should be directed to Dr. Kevin Murnane (kevin.murnane@lsuhs.edu).
