# Supplementary material for: Fronto-striatal neurometabolite signatures of impulsivity in early abstinence from methamphetamine
Source: Neuroimage Clin. 2026 Jun 1;50:104015. doi: 10.1016/j.nicl.2026.104015 (PMC13265701; doi:10.1016/j.nicl.2026.104015)
Supplement: Supplementary Data 1 [file mmc1.docx]

**SUPPLEMENTARY MATERIALS**

**Fronto-Striatal Neurometabolite Signatures of Impulsivity in Early Abstinence from Methamphetamine**

Alexandru Mihai Dumitrescu^a,b^, M. Frances Vest^a,b^, Jennifer M. Loftis^c,d,e^ and Kevin S. Murnane^a,b,f,g^

^a^ Louisiana Addiction Research Center, Louisiana State University Health Sciences Center at Shreveport, Shreveport, LA, United States

^b^ Department of Pharmacology, Toxicology & Neuroscience, Louisiana State University Health Sciences Center at Shreveport, Shreveport, LA, United States

^c^ Research & Development Service, Veterans Affairs Portland Health Care System, 3710 SW U.S. Veterans Hospital Rd., Portland, Oregon, United States

^d^ Department of Psychiatry, Center for Mental Health Innovation, Oregon Health & Science University, 3181 SW Sam Jackson Park Rd, Portland, Oregon, United States

^e^ Department of Behavioral Neuroscience, Oregon Health & Science University, 3181 SW Sam Jackson Park Rd, Portland, Oregon, United States

^f^ Center for Brain Health, Louisiana State University Health Sciences Center at Shreveport, Shreveport, LA, United States

^g^ Department of Psychiatry and Behavioral Medicine, Louisiana State University Health Sciences Center at Shreveport, Shreveport, LA, United States

**Corresponding author**:

Kevin S. Murnane, Ph.D.

Associate Professor, Department of Pharmacology, Toxicology & Neuroscience

Associate Professor, Department of Psychiatry

Director of Basic Sciences Research, Louisiana Addiction Research Center

Louisiana State University Health Sciences Center – Shreveport

1501 Kings Highway, Shreveport, LA, 71103

E-mail: [kevin.murnane@lsuhs.edu](mailto:kevin.murnane@lsuhs.edu)


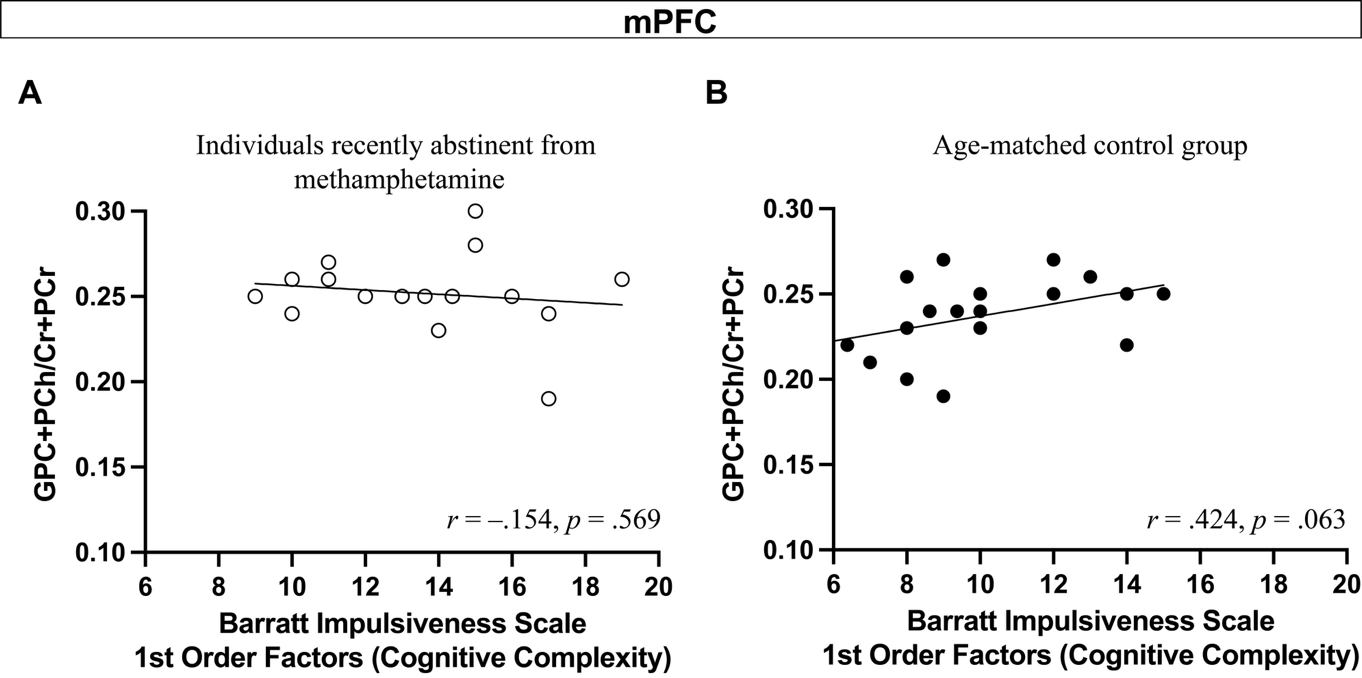


**Supplementary Figure 1.** Relationship between mPFC GPC+PCh/Cr+PCr ratio and BIS-11 Cognitive Complexity subscale scores by group. **A.** In individuals recently abstinent from methamphetamine, no significant association was observed. **B.** In the age-matched controls, a positive association was observed, although it did not reach statistical significance.

| **Neurometabolite** | **Population** | **Region** | **N** | **Mean** ± SD |
| --- | --- | --- | --- | --- |
| **Cr+PCr** | Age-matched controls | Right DLPFC | 21 | 0.000189 ± 0.000011 |
|  |  | Left DLPFC | 21 | 0.000176 ± 0.000018 |
|  |  | ACC | 21 | 0.000168 ± 0.000036 |
|  |  | CAUD | 21 | 0.000138 ± 0.000029 |
|  |  | mPFC | 21 | 0.000178 ± 0.000019 |
|  | Individuals recently abstinent from methamphetamine | Right DLPFC | 20 | 0.000186 ± 0.000018 |
|  |  | Left DLPFC | 20 | 0.000177 ± 0.000022 |
|  |  | ACC | 20 | 0.000182 ± 0.000042 |
|  |  | CAUD | 20 | 0.000142 ± 0.000022 |
|  |  | mPFC | 20 | 0.000157 ± 0.000041 |
| **Cr** | Age-matched controls | Right DLPFC | 21 | 0.000095 ± 0.000015 |
|  |  | Left DLPFC | 21 | 0.000083 ± 0.000015 |
|  |  | ACC | 21 | 0.000073 ± 0.000034 |
|  |  | CAUD | 21 | 0.000066 ± 0.000028 |
|  |  | mPFC | 21 | 0.000078 ± 0.000022 |
|  | Individuals recently abstinent from methamphetamine | Right DLPFC | 20 | 0.000105 ± 0.000025 |
|  |  | Left DLPFC | 20 | 0.000079 ± 0.000016 |
|  |  | ACC | 20 | 0.000071 ± 0.000045 |
|  |  | CAUD | 20 | 0.000088 ± 0.000034 |
|  |  | mPFC | 20 | 0.000060 ± 0.000032 |
| **PCr** | Age-matched controls | Right DLPFC | 21 | 0.000051 ± 0.000006 |
|  |  | Left DLPFC | 21 | 0.000048 ± 0.000007 |
|  |  | ACC | 21 | 0.000043 ± 0.000012 |
|  |  | CAUD | 21 | 0.000042 ± 0.000009 |
|  |  | mPFC | 21 | 0.000042 ± 0.000007 |
|  | Individuals recently abstinent from methamphetamine | Right DLPFC | 20 | 0.000050 ± 0.000006 |
|  |  | Left DLPFC | 20 | 0.000046 ± 0.000008 |
|  |  | ACC | 20 | 0.000048 ± 0.000020 |
|  |  | CAUD | 20 | 0.000047 ± 0.000008 |
|  |  | mPFC | 20 | 0.000039 ± 0.000012 |
| **GPC+PCh** | Age-matched controls | Right DLPFC | 21 | 0.000096 ± 0.000017 |
|  |  | Left DLPFC | 21 | 0.000093 ± 0.000021 |
|  |  | ACC | 21 | 0.000096 ± 0.000034 |
|  |  | CAUD | 21 | 0.000071 ± 0.000028 |
|  |  | mPFC | 21 | 0.000099 ± 0.000019 |
|  | Individuals recently abstinent from methamphetamine | Right DLPFC | 20 | 0.000081 ± 0.000018 |
|  |  | Left DLPFC | 20 | 0.000099 ± 0.000023 |
|  |  | ACC | 20 | 0.000111 ± 0.000052 |
|  |  | CAUD | 20 | 0.000054 ± 0.000032 |
|  |  | mPFC | 20 | 0.000094 ± 0.000047 |

**Supplementary Table 1. Absolute neurometabolite estimates by group and ROI.** Mean (± SD) LCModel-estimated absolute concentrations of Cr+PCr, Cr, PCr, and GPC+PCh for each region of interest (ROI) in individuals recently abstinent from methamphetamine and age-matched controls. Estimates are reported in LCModel institutional units (software-defined scaling). Columns display the sample size (N), mean, and standard deviation (SD) for each Population × ROI combination. *Cr+PCr, creatine plus phosphocreatine; Cr, creatine; PCr, phosphocreatine; GPC+PCh, total choline; ROI, region of interest; Right DLPFC, right dorsolateral prefrontal cortex; Left DLPFC, left dorsolateral prefrontal cortex; ACC, anterior cingulate cortex; CAUD, caudate nucleus; mPFC, medial prefrontal cortex; SD, standard deviation.*

| **Neurometabolite** | **Effect** | **Num *df*** | **Den *df*** | **F-value** | ***p*-value** |
| --- | --- | --- | --- | --- | --- |
| **Cr+PCr** | Population | 1 | 39 | 0.04 | 0.842 |
|  | Region | 4 | 156 | 26.178 | < .001 |
|  | Population × Region | 4 | 156 | 3.246 | 0.014 |
| **Cr** | Population | 1 | 39 | 0.117 | 0.734 |
|  | Region | 4 | 156 | 7.588 | < .001 |
|  | Population × Region | 4 | 156 | 3.098 | 0.017 |
| **PCr** | Population | 1 | 33.853 | 0.128 | 0.723 |
|  | Region | 4 | 53.117 | 6.464 | < .001 |
|  | Population × Region | 4 | 53.117 | 1.812 | 0.140 |
| **GPC+PCh** | Population | 1 | 39 | 0.553 | 0.462 |
|  | Region | 4 | 156 | 10.652 | < .001 |
|  | Population × Region | 4 | 156 | 2.025 | 0.093 |

**Supplementary Table 2. Linear mixed-effects ANOVA** **for absolute Cr+PCr, Cr, PCr, and GPC+PCh.** Results of linear mixed-effects (LME) models testing the effects of Population (individuals recently abstinent from methamphetamine vs. age-matched controls), Region (five ROIs), and their interaction on LCModel-estimated absolute concentrations of Cr+PCr, Cr, PCr, and GPC+PCh. Each model included fixed effects of Population, Region, and the Population × Region interaction, with a random intercept for Subject to account for repeated measurements across regions. Estimates are reported in LCModel institutional units (software-defined scaling). The table reports numerator degrees of freedom (Num *df*), denominator degrees of freedom (Den *df*), F-statistics, and associated *p-*values for each effect. *Cr+PCr, creatine plus phosphocreatine; Cr, creatine; PCr, phosphocreatine; GPC+PCh, total choline; ROIs, regions of interest.*

| **Region** | **Population** | **N** | **Mean ±** **SEM** | **Median CRLB** | **Range** | **Shapiro-Wilk (*p-*value)** |
| --- | --- | --- | --- | --- | --- | --- |
| **Right DLPFC** | Individuals recently abstinent from methamphetamine | 20 | 6.00 ± 0.22 | 6 | 4-7 | 0.003476 |
|  | Age-matched controls | 21 | 6.33 ± 0.17 | 6 | 4-7 | 0.000126 |
| **Left DLPFC** | Individuals recently abstinent from methamphetamine | 20 | 7.10 ± 0.41 | 7 | 4-10 | 0.188214 |
|  | Age-matched controls | 21 | 6.81 ± 0.16 | 7 | 6-8 | 0.000730 |
| **ACC** | Individuals recently abstinent from methamphetamine | 20 | 7.25 ± 0.62 | 6.5 | 5-14 | 0.000233 |
|  | Age-matched controls | 21 | 6.95 ± 0.45 | 6 | 5-14 | 0.000016 |
| **CAUD** | Individuals recently abstinent from methamphetamine | 20 | 7.30 ± 0.55 | 7 | 1-11 | 0.120087 |
|  | Age-matched controls | 21 | 7.29 ± 0.33 | 7 | 5-11 | 0.087388 |
| **mPFC** | Individuals recently abstinent from methamphetamine | 20 | 7.25 ± 0.50 | 6 | 5-14 | 0.000051 |
|  | Age-matched controls | 21 | 6.38 ± 0.28 | 6 | 5-11 | 0.000049 |

**Supplementary Table 3. Summary of Glu+Gln CRLB by region and group. For each region of interest (ROI), the table reports the number of observations (N), mean ± SEM Glu+Gln Cramer–Rao lower bounds (CRLB, %), median, range, and Shapiro–Wilk *p‑*value for normality in individuals recently abstinent from methamphetamine and age‑matched controls. Across ROIs and groups, mean Glu+Gln CRLB values were approximately 6–7%, with medians of 6–7% and ranges typically 4–11%.** *Right DLPFC, right dorsolateral prefrontal cortex; Left DLPFC, left dorsolateral prefrontal cortex; ACC, anterior cingulate cortex; CAUD, caudate nucleus; mPFC, medial prefrontal cortex.*

| **Region** | **Individuals recently abstinent from methamphetamine**  **(N = 20)** | **Age-matched controls**  **(N = 21)** | **Individuals recently abstinent from methamphetamine**  **vs.**  **Age-matched controls** | |
| --- | --- | --- | --- | --- |
|  | **Mean ± SEM** | **Mean ± SEM** | **Independent samples t-test**  **(*p-*value)** | **Wilcoxon rank sum**  **(*p-*value)** |
| **Right DLPFC** | 6 ± 0.22 | 6.33 ± 0.17 | 0.24 | 0.26 |
| **Left DLPFC** | 7.1 ± 0.41 | 6.81 ± 0.16 | 0.52 | 0.63 |
| **ACC** | 7.25 ± 0.62 | 6.95 ± 0.45 | 0.70 | 0.88 |
| **CAUD** | 7.3 ± 0.55 | 7.29 ± 0.33 | 0.98 | 0.88 |
| **mPFC** | 7.25 ± 0.50 | 6.38 ± 0.28 | 0.14 | 0.26 |

**Supplementary Table 4.** **Group comparisons of Glu+Gln CRLB by region.** For each region of interest (ROI), the table reports the mean ± SEM Cramér–Rao lower bounds (CRLB, %) for Glu+Gln and the sample size per group in individuals recently abstinent from methamphetamine and age-matched controls. Between-group differences were evaluated using independent-samples t-tests and Wilcoxon rank-sum tests. *Right DLPFC, right dorsolateral prefrontal cortex; Left DLPFC, left dorsolateral prefrontal cortex; ACC, anterior cingulate cortex; CAUD, caudate nucleus; mPFC, medial prefrontal cortex*

| **Region** | **Population** | **N** | **Outliers (IQR method)** | **Outliers (\|z\| > 2.5)** | **Outliers (\|z\| > 3)** | **CRLB > 15% (N)** |
| --- | --- | --- | --- | --- | --- | --- |
| **Right DLPFC** | Individuals recently abstinent from methamphetamine | 20 | 0 | 0 | 0 | 0 |
|  | Age-matched controls | 21 | 1 | 1 | 0 | 0 |
| **Left DLPFC** | Individuals recently abstinent from methamphetamine | 20 | 0 | 0 | 0 | 0 |
|  | Age-matched controls | 21 | 0 | 0 | 0 | 0 |
| **ACC** | Individuals recently abstinent from methamphetamine | 20 | 3 | 0 | 0 | 0 |
|  | Age-matched controls | 21 | 2 | 1 | 1 | 0 |
| **CAUD** | Individuals recently abstinent from methamphetamine | 20 | 1 | 1 | 0 | 0 |
|  | Age-matched controls | 21 | 0 | 0 | 0 | 0 |
| **mPFC** | Individuals recently abstinent from methamphetamine | 20 | 4 | 1 | 1 | 0 |
|  | Age-matched controls | 21 | 1 | 1 | 1 | 0 |

**Supplementary Table 5. Outlier flags for Glu+Gln CRLB.** For each region of interest (ROI) and population group, the table reports the number of valid observations (N) and the number of values flagged as potential outliers using three criteria: the interquartile range (IQR) rule, |z| > 2.5, and |z| > 3. The final column indicates the number of observations exceeding the CRLB quality threshold (>15%). *Right DLPFC, right dorsolateral prefrontal cortex; Left DLPFC, left dorsolateral prefrontal cortex; ACC, anterior cingulate cortex; CAUD, caudate nucleus; mPFC, medial prefrontal cortex*


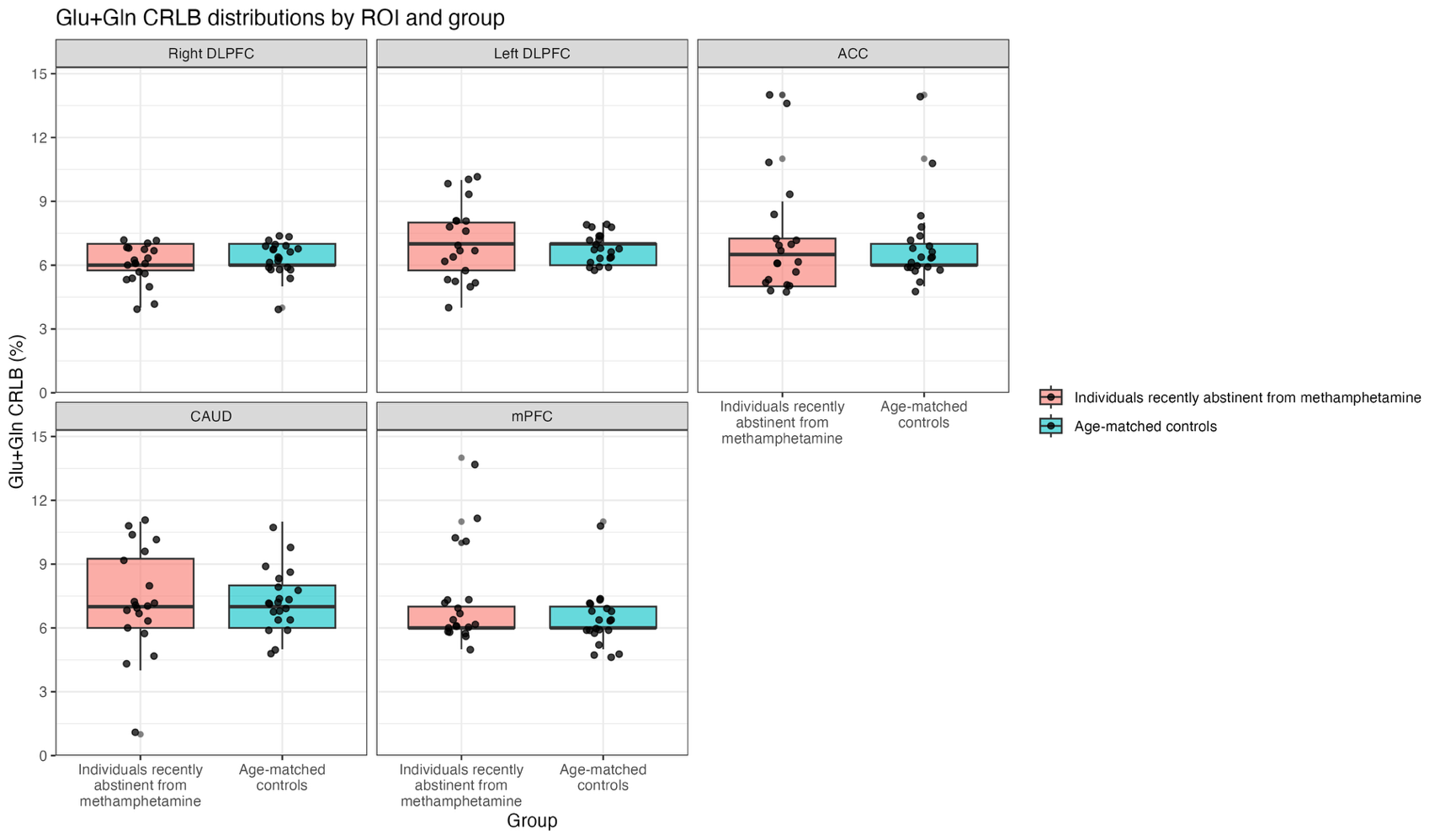


**Supplementary Figure 2. Glu+Gln CRLB distributions by region and group**. Boxplots with jittered individual data points illustrate the distribution of Glu+Gln Cramér–Rao lower bounds (CRLB, %) in individuals recently abstinent from methamphetamine and age-matched controls across the right and left dorsolateral prefrontal cortex (DLPFC), anterior cingulate cortex (ACC), caudate (CAUD), and medial prefrontal cortex (mPFC). Distributions are compact and largely overlapping between groups in all ROIs, with typical CRLB values around 6–7% and no cluster of high-CRLB estimates.

**Supplementary Table 6.** Intercorrelations and multicollinearity diagnostics for covariates.

| **Group** | **Covariate pair** | ***r*** | ***p-*value** |
| --- | --- | --- | --- |
| Individuals recently abstinent from methamphetamine | Educational level – Depression | −0.021 | 0.93 |
|  | Educational level – Anxiety | −0.062 | 0.796 |
|  | Educational level – Stress | −0.207 | 0.38 |
|  | Depression – Anxiety | 0.769 | <0.001 |
|  | Depression – Stress | 0.933 | <0.001 |
|  | Anxiety – Stress | 0.777 | <0.001 |
| Age-matched controls | Educational level – Depression | −0.245 | 0.299 |
|  | Educational level – Anxiety | −0.126 | 0.595 |
|  | Educational level – Stress | −0.176 | 0.459 |
|  | Depression – Anxiety | 0.662 | 0.001 |
|  | Depression – Stress | 0.632 | 0.003 |
|  | Anxiety – Stress | 0.71 | <0.001 |

**A.** Pearson correlations between educational level and DASS-42 subscales by group. Values are Pearson correlation coefficients (r) with two‑tailed *p‑*values. Education is coded as an ordinal variable representing years/level of education. Depression, Anxiety, and Stress are DASS‑42 subscale scores.

| **Covariate** | **Tolerance (min–max)** | **VIF (min–max)** |
| --- | --- | --- |
| Educational level | 0.594 – 0.718 | 1.39 – 1.69 |
| Depression | 0.087 – 0.109 | 9.22 – 11.55 |
| Anxiety | 0.337 – 0.454 | 2.20 – 2.96 |
| Stress | 0.081 – 0.094 | 10.61 – 12.28 |

**B.** Variance inflation factors (VIF) and tolerance for covariates included in neurometabolite LME models. Tolerance and VIF values are summarized across all LME models used in the neurometabolite analyses; ranges reflect minimum and maximum values observed across regions of interest (ROIs). Tolerance < 0.10 and VIF > 10 indicate substantial multicollinearity.

**Supplementary Table 7.** Sensitivity analyses using educational level and negative affect composite as covariates.

| **Subscale** | **Group** | **Adjusted mean** | **SE** | **95% CI lower** | **95% CI upper** | **Mean difference (groups)** | ***p-*value** |
| --- | --- | --- | --- | --- | --- | --- | --- |
| Attention | Individuals recently abstinent from methamphetamine | 10.421 | 0.632 | 9.175 | 11.668 | 0.393 | 0.659 |
|  | Age-matched controls | 10.028 | 0.614 | 8.818 | 11.239 |  |  |
| Cognitive Instability | Individuals recently abstinent from methamphetamine | 6.843 | 0.632 | 5.596 | 8.089 | −0.821 | 0.360 |
|  | Age-matched controls | 7.664 | 0.616 | 6.449 | 8.879 |  |  |
| Motor | Individuals recently abstinent from methamphetamine | 18.527 | 0.632 | 17.280 | 19.773 | 3.563 | <0.001 |
|  | Age-matched controls | 14.964 | 0.616 | 13.749 | 16.179 |  |  |
| Perseverance | Individuals recently abstinent from methamphetamine | 8.316 | 0.632 | 7.070 | 9.563 | 0.452 | 0.614 |
|  | Age-matched controls | 7.864 | 0.616 | 6.649 | 9.079 |  |  |
| Self‑Control | Individuals recently abstinent from methamphetamine | 13.211 | 0.632 | 11.964 | 14.457 | 0.997 | 0.266 |
|  | Age-matched controls | 12.214 | 0.616 | 10.999 | 13.429 |  |  |
| Cognitive Complexity | Individuals recently abstinent from methamphetamine | 12.369 | 0.632 | 11.122 | 13.615 | 1.905 | 0.034 |
|  | Age-matched controls | 10.464 | 0.616 | 9.249 | 11.679 |  |  |

**A.** Mixed‑design ANCOVA on BIS‑11 first‑order subscales (covariates: educational level + negative affect composite). Adjusted marginal means (± SE) and 95% confidence intervals for BIS-11 first-order subscale scores are shown for individuals recently abstinent from methamphetamine and age-matched controls. Estimates are derived from a mixed-design ANCOVA with Population as the between-subjects factor, BIS-11 first-order subscales as the within-subjects factor, and educational level plus the negative affect composite as covariates. p-values represent Bonferroni-adjusted pairwise comparisons between groups (individuals recently abstinent from methamphetamine - age-matched controls).

| **Outcome (group, ROI)** | **Predictors in model*** | **Key neurometabolite predictor (ROI)** | **β (primary model)** | **p (primary)** | **β (composite model)** | **p (composite)** |
| --- | --- | --- | --- | --- | --- | --- |
| Self‑Control (age-matched controls, CAUD) | mI/Cr+PCr, GPC+PCh/Cr+PCr, group, educational level, affect covariates | CAUD mI/Cr+PCr | 0.529 | 0.017 | 0.585 | 0.060 |
| Self‑Control (individuals recently abstinent from methamphetamine, CAUD) |  |  | −0.355 | 0.162 | −0.332 | 0.421 |
| Cognitive Complexity (controls, mPFC) | GPC+PCh/Cr+PCr, group, educational level, affect covariates | mPFC GPC+PCh/Cr+PCr | 0.424 | 0.063 | 0.288 | 0.247 |
| Cognitive Complexity (methamphetamine, mPFC) |  |  | −0.154 | 0.569 | −0.072 | 0.807 |

**B.** Neurometabolite–impulsivity regressions (key models). Regression coefficients for key neurometabolite predictors are shown for models including educational level and affective covariates. Regression coefficients for key neurometabolite predictors are shown for models including educational level and affective covariates. *Predictors in each model include the listed neurometabolite(s), population group, educational level, and affective covariates (either separate DASS‑42 depression, anxiety, and stress scores in the primary models or the negative affect composite in the composite models). Under the composite specification, previously observed or trend-level associations between Self-Control and CAUD mI/Cr+PCr in controls and between Cognitive Complexity and mPFC GPC+PCh/Cr+PCr in controls are attenuated and no longer statistically significant (*p* ≥ 0.06). However, the overall pattern of group and MRS effects remains similar. *P-*values are Holm–Bonferroni–corrected within the family of neurometabolite–impulsivity regression analyses.

**Supplementary Table 8.** Exploratory sensitivity analyses examining associations between abstinence duration and behavioral or neurometabolite measures within the methamphetamine group.

| **Measure** | **N** | **r** | ***p*-value** |
| --- | --- | --- | --- |
| Motor | 20 | -0.096 | 0.697 |
| Self-Control | 20 | 0.052 | 0.833 |
| Cognitive Complexity | 20 | 0.076 | 0.756 |

**A.** Exploratory Pearson correlation analyses examining associations between abstinence duration and BIS-11 first-order subscales that showed significant group differences in the primary analyses. *The table reports Pearson correlation coefficients (r), sample size (N), and associated p-values for each analysis.* *BIS-11, Barratt Impulsiveness Scale-11; N, number of participants.*

| **ROI-specific neurometabolite ratios** | **N** | **r** | ***p*-value** |
| --- | --- | --- | --- |
| CAUD mI/Cr+PCr | 20 | -0.128 | 0.625 |
| CAUD GPC+PCh/Cr+PCr | 20 | -0.355 | 0.163 |
| mPFC GPC+PCh/Cr+PCr | 20 | -0.24 | 0.39 |

**B.** Exploratory Pearson correlation analyses examining associations between abstinence duration and ROI-specific neurometabolite ratios that showed significant group differences in the primary analyses. *The table reports Pearson correlation coefficients (r), sample size (N), and associated p-values for each analysis.* *CAUD, caudate nucleus; mPFC, medial prefrontal cortex; mI, myo-inositol; GPC+PCh, total choline; Cr+PCr, creatine plus phosphocreatine; N, number of participants.*
